# Comparison of Aqueous and 1-Octanol Solubility as well as Liquid–Liquid Distribution of Acyclovir Derivatives and Their Complexes with Hydroxypropyl-β-Cyclodextrin

**DOI:** 10.1007/s10953-013-9995-8

**Published:** 2013-04-26

**Authors:** Małgorzata Koźbiał, Paweł Gierycz

**Affiliations:** Institute of Physical Chemistry, Polish Academy of Sciences, Kasprzaka 44/52, 01-224 Warsaw, Poland

**Keywords:** Acyclovir derivatives, Solubility, Cyclodextrin, Complexation, Partition

## Abstract

The aim of the presented work is the comparison of aqueous and 1-octanol solubilities of different acyclovir derivatives and their hydroxypropyl-β-cyclodextrin inclusion complexes. The solubility measurements were carried out at different temperatures over the range 25–45 °C using water, 1-octanol, water saturated with 1-octanol, 1-octanol saturated with water, buffered aqueous solutions (pH = 5.5 and 7.0) and buffered aqueous solutions containing cyclodextrin as solvents. The aqueous solubilities of the compounds are very low but may be enhanced by complexation with hydroxypropyl-β-cyclodextrin, especially if the acyclovir derivatives have aromatic groups which may be included in the cyclodextrin cavity. The values of 1-octanol–water partition coefficients of acyclovir derivatives, obtained using extraction experiments, showed a similar sequence as the solubility results in 1-octanol. Additionally, some molecular mechanics and molecular dynamic calculations were performed to determine optimized structures of acyclovir derivative complexes with β-cyclodextrin treated as a model.

## Introduction

Herpes viruses are the most wide-spread DNA human viruses, so searching for new efficient drugs possessing good antiviral activity is very important. The antiviral drugs have been used from 1960s, but new drugs with higher bioactivity and lower toxicity are continually being developed and tested. The first antiviral drugs: synthetic analogs of pyrimidine–idoxyuridine, trifluridine and vidarabine, were synthesized in 1960 [1]. They have low bioactivity and high toxicity, so now are used only to cure eye infections. Acyclovir, **1,** which is a substituted guanine, belongs to the next generation of antiviral drugs. This drug, synthesized in 1977 by Ellion and coworkers [2], was registered in 1982 and now is a well known, selective, antiviral agent [1]. The toxicity of acyclovir is lower than other antiviral dugs, due to its acyclic sugar chain. Acyclovir, after phosphorylation of the sugar chain, which takes place in infected cells, can inhibit replication of viruses HSV-1 and HSV-2. Several derivatives of acyclovir, obtained by modification of acyclic chain, have been synthesized and registered as drugs [1, 3]. The mechanism of their antiviral action is similar to that of acyclovir. Other examples of acyclovir derivatives registered as antiviral drugs are prodrugs, which are bioactive only after disintegration to acyclovir in the human body. Application of antiviral drugs as acyclovir prodrugs, like valacyclovir or famciclovir, gives the possibility of enhancing the absorption of acyclovir in the human body. In the case of application of valacyclovir, absorption is four times higher than by application of acyclovir [1].

**Fig. 1:**
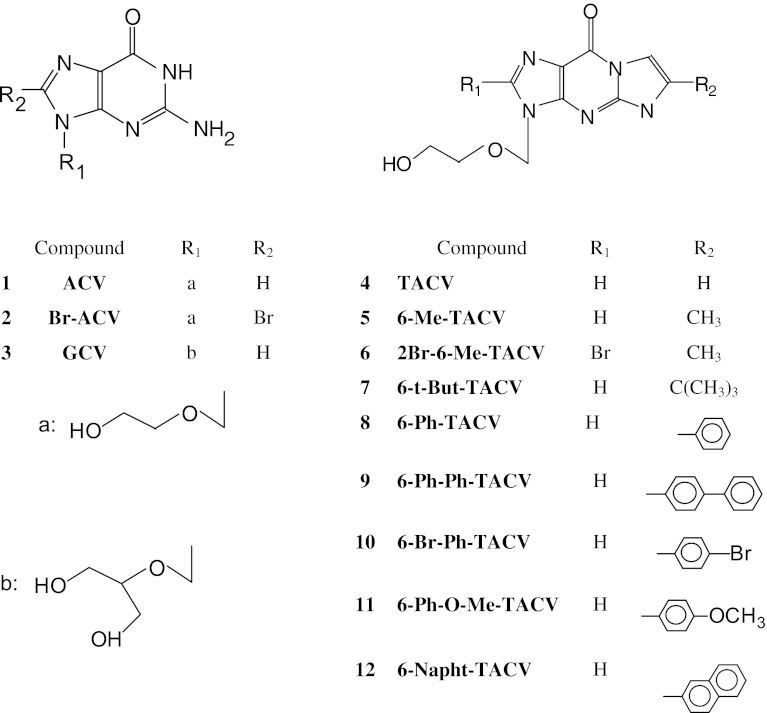
Structures of acyclovir and its derivatives which are the subject of this work

Some guanine substituted acyclovir derivatives were synthesized by De Clerq and coworkers [4, 5]. Those derivatives have somewhat greater antiviral activity towards HSV viruses than the parent drug. Tricyclic acyclovir derivatives synthesized by Golankiewicz and coworkers have high antiviral activity and good fluorescent properties, important for monitoring their concentration in body fluids. Their synthesis, structures, antiviral and spectral properties have been the subject of many publications [6–14]. The bioactivity and spectral properties of tricyclic acyclovir derivatives were summarized by Golankiewicz and Ostrowski in a mini review [15]; they found that, generally, substitution in appended rings enhanced the antiviral properties and selectivity of the studied compounds. The magnitude of antiviral activity towards different viruses depends on the type, position of substituted group and the kind of acyclic sugar chain. In particular, substitution in the 6th position by a phenyl or 4-biphenylyl group afforded the greatest increase in antiviral activity [15]. Compound **11** (6-Ph-O-Me-TACV) (Fig. 1) shows 2–7 times higher antiherpetic activity towards HSV-1 and HSV-2 viruses than the parent acyclovir when comparing the concentrations required to reduce virus-induced cytopathicity by 50 % [8].

In our earlier work [16] we determined aqueous and 1-octanol solubility and partition coefficient between these two solvents of acyclovir and chosen acyclovir derivatives. Structures of those compounds are shown in Fig. 1. The work showed extremely low aqueous solubilities (10^−3^ to 10^−5^ mol·dm^−3^) for the main group of these compounds, especially with aromatic substituents. Other thermodynamic properties of these compounds in aqueous solutions, e.g. partial molar volumes and heat capacities, were determined by Zielenkiewicz and coworkers [17, 18]. The low aqueous solubility of tricyclic acyclovir derivatives is disadvantageous for drug application and absorption by cells, so enhancement of their solubility seems to be very important. One of the ways of enhancing solubility of tricyclic analogs of acyclovir in aqueous solutions can be the formation of inclusion complexes with cyclodextrins. Natural and substituted cyclodextrins are known as solubilizing agents for many drugs used in pharmacy, acting by forming inclusion and non-inclusion complexes with them [19]. Native and substituted cyclodextrins are generally nontoxic and may be used in pharmacy, mainly as solubilizers, but sometimes as stabilizers or in order to enhance bioactivity or reduce local drug irritation [18]. Hydroxypropyl-derivatives of α-, β-, and γ-cyclodextrins are especially suitable for drug formulation due to their amorphous form in the solid state. Now, worldwide more than 40 different drugs are marketed in the form of native and substituted cyclodextrin complexes.

Our study on complexation of chosen tricyclic acyclovir derivatives in aqueous buffered solutions indicates significant enhancement of solubility of the studied compounds, especially these possessing aromatic substituents [20]. The increase of solubility is mainly caused by inclusion of hydrophobic groups inside the cyclodextrin cavity. The complexation of alkyl and aromatic parts of tricyclic acyclovir derivatives in cyclodextrin cavities was confirmed by ^1^H NMR. Differential scanning calorimetry (DSC) experiments also indicate the existence of cyclodextrin complexes with studied compounds in the solid state [19].

In the literature, there are only a few reports of acyclovir and their derivatives complexed with cyclodextrins [21–26]. Stability constants of these complexes and the enhancement of aqueous solubility are low, but results of in vitro experiments indicated that stability, absorption and permeation of drugs used as cyclodextrin complexes are higher than those of the pure drugs [25, 26].

The aim of our work is the comparison of aqueous and 1-octanol solubilities of tricyclic acyclovir derivatives possessing high antiviral activity and their cyclodextrin complexes, and connecting these results with the values of their partition coefficients between water and 1-octanol determined by extraction experiments. Additionally, molecular modeling and molecular dynamic optimizations of cyclodextrin inclusion complexes with tricyclic acyclovir analogs were performed in order to explain structures and the physicochemical properties of the complexes.

## Experimental

### Materials

The following compounds (**1**–**12**) synthesized by Golankiewicz and coworkers [6–15] were investigated in our work: **1,** acyclovir, 9-[(2-hydroxyethoxy)methyl] guanine (ACV); **2,** 8-bromo-9-[(2-hydroxyethoxy)methyl] guanine (8-Br-ACV); **3,** ganciclovir, 9-[(1,3-dihydroxy-2-propoxy)methyl]guanine (GCV); **4,** 3,9-dihydro-3-[(2-hydroxyethoxy)methyl]-9-oxo-5H-imidazol[1,2-a]purine (TACV); and its substituted derivatives; **5,** 6-methyl (6-Me-TACV); **6,** 2-bromo-6-methyl (2-Br-6-Me-TACV); **7,** 6-tert-butyl (6-t-But-TACV); **8,** 6-phenyl (6-Ph-TACV); **9**, 6-(4-biphenylyl) (6-Ph-Ph-TACV); **10**, 6-(4-bromophenyl) (6-Br-Ph-TACV); **11**, 6-(2-naphthyl) (6-napht)-TACV]; **12**, 6-(4-methoxyphenyl), (6-Me-O-Ph-TACV).

The structures of these compounds are given in Fig. 1. All of the compounds were freshly prepared for these studies. They were purified by recrystallization and the resulting compounds were homogenous according to HPLC and ^1^H NMR analysis.

The hydroxypropyl-β-cyclodextrin (HP-β-CD) was purchased from Janssen Drug Delivery System (USA), R81216 no 30 221 54, and used without additional purification. KH_2_PO_4_ and Na_2_HPO_4_ salts used for preparation of buffer solutions were of analytical grade (POCh, Gliwice, Poland). Distilled and deionized water from a Milli-Q system (Millipore, USA) was used for solution preparation.

### Solubility Experiments

#### Determination of Solubility in Water or 1-Octanol

Saturated solutions of compounds **1**–**12** were obtained in a thermostated apparatus holding glass ampoules of 15 cm^3^ volume, which could be rotated by 180°. The sample of substance examined (10 mg or 80 mg for 6-Me-TACV) was placed in a glass ampoule and double-distilled, deionized water (Millipore, Elix 5 purifier), or pure anhydrous 1-octanol (Sigma 99 %) was added. To attain thermodynamic equilibrium, the solute and the solvent were mixed continuously for a minimum of 4 days at a speed of 25 rpm. After the experiment was completed, the sample was centrifuged at 40,000 rpm for 40 min at 25 ± 0.1 °C, the supernatant liquid was collected, diluted, and its concentration determined with a UV–Vis (Varian Carry IE) spectrophotometer. The experiments were repeated at least three times for each solubility.

#### Determination of Solubility in Buffered Cyclodextrin Solutions

Solubilities of the studied compounds was ensured according to the excess method of Higuchi and Connors [30]. Compounds **4**–**9** and **11**–**12** were chosen for these studies, because their aqueous solubility is very low and should be enhanced for pharmaceutical applications. Additionally, their structures suggested that these compounds may be come complexed by cyclodextrins, especially tricyclic acyclovir derivatives possessing aromatic substituents. An excess amount of compounds **4**–**9** and **11**–**12** was added to 1.5 mL of aqueous phosphate buffer (concentration of buffer was equal 0.067 mol·dm^−3^) solutions, (pH = 5.5 or 7.0) containing various concentration of HP-β-CD. The cyclodextrin concentration changed in the range of 10^−3^ to 2 × 10^−2^ mol·dm^−3^. The suspensions were shaken in screw-capped plastic vials in a water bath thermostat with the volume of 60 dm^3^ (home made apparatus by IChF PAN) at 25 and 37 °C. After 7 days equilibrium was reached and then the suspensions were centrifuged and the absorbance of the studied compounds was measured by UV–Vis spectrophotometry using a Shimadzu UV 2401 PC Spectrometer equipped with a thermostatically controlled cell compartment. During the measurement of absorbance, a buffered-solution of HP-β-CD, of the same concentration as in the studied solution, was always put as the reference. The average values of at least three independent determinations of the absorbance were used for calculation of the concentrations of the particular acyclovir derivatives.

### Extraction Experiments

The equipment described above was also used for the determination of the 1-octanol-water partition coefficients. The procedure was as follows:

- An aqueous solution at a concentration of one half of the solubility of the substance examined was prepared.
- The solution was placed in an ampoule and an identical volume of 1-octanol was added.
- The ampoule was placed in a thermostat.
- The measurement was continued for 2 days with continuous stirring.
- The initial concentration in the aqueous solution and the final concentration of the substance after each measurement were determined by spectrophotometry.

The partition coefficient was evaluated as:

1 $$ P_{{{\text{oc}}/{\text{w}}}} = \frac{{C_{\text{oc}} }}{{C_{\text{w}} }} $$

where *C*_oc_ and *C*_w_ are the molar concentrations of the solute in the 1-octanol and water phases, respectively. The correctness of the *P*_oc*/*w_ value of each compound was verified by checking the mass balance of the starting amount of the substance and the total amount of the substance partitioned between the two phases.

### Molecular Mechanics and Molecular Dynamics Experiments

Calculations were performed with the OPLS force field using HyperChem 7.0 software. Molecular mechanic and molecular dynamic simulations were made using the conjugate gradient algorithm included in HyperChem 7.0 software with convergence criteria of 0.004 kJ·mol^−1^. For each complex energy optimization 100 cycles of molecular mechanic and molecular dynamic calculations were performed. Then 10 optimized structures of complexes with the lowest energy of complexation were taken and average values of energy of complexation and surfaces of acyclovir derivative and cyclodextrin were determined. Calculations were performed with β-cyclodextrin because hydroxypropyl-β-cyclodextrin used in experiments doesn’t have an exactly defined structure. Simulations started from crystallographic structures of β-cyclodextrin and acyclovir derivatives taken from the Cambridge Data Base of crystallographic structures: β-cyclodextrin CCDC ID: BUVSEQ02 [27], and acyclovir derivatives CCDC ID:QOGQOS [28].

## Results and Discussion

**Fig. 2:**
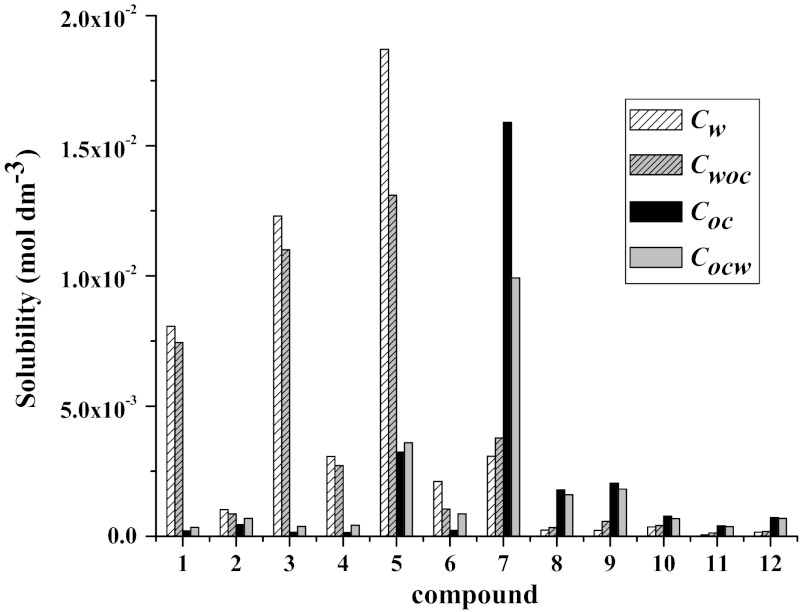
Solubility of compounds **1**–**12** in water (*C*_w_), water saturated with 1-octano(*C*_woc_), 1-octanol (*C*_oc_) and 1-octanol saturated with water (*C*_ocw_)

**Fig. 3:**
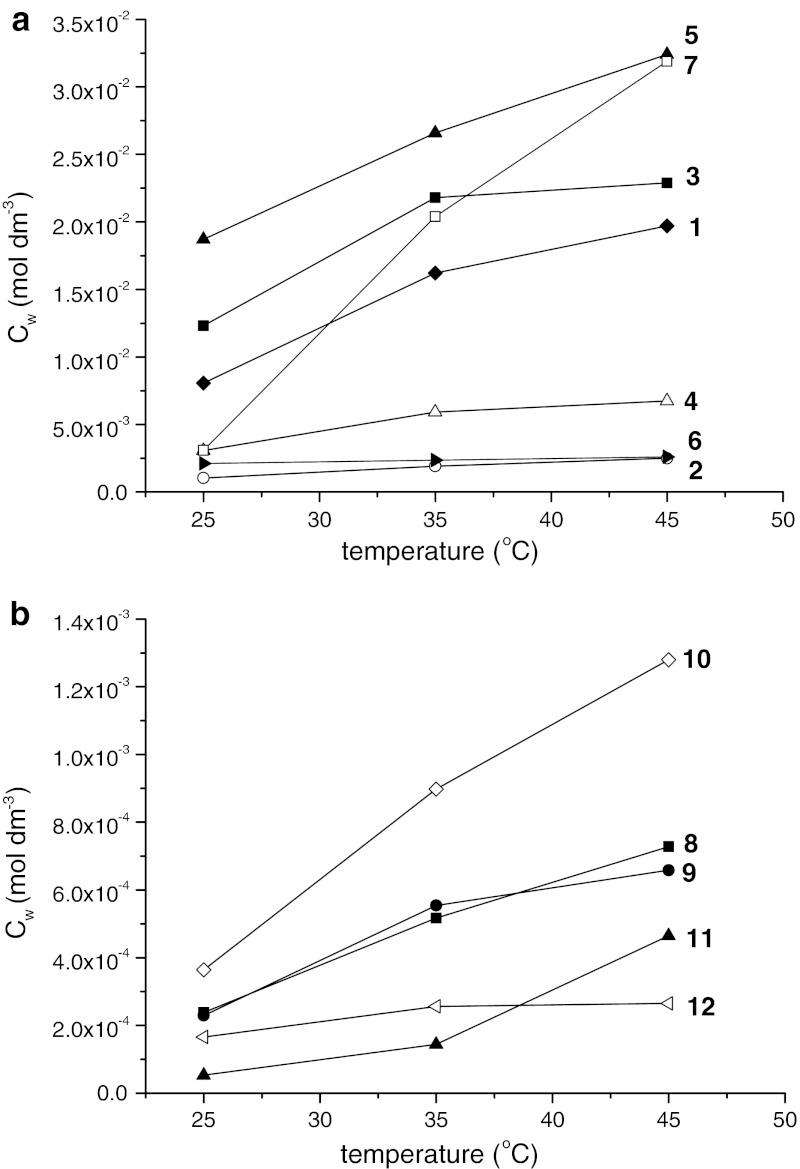
Solubility of compounds **1**–**12** versus temperature

Acyclovir derivatives which are the subject of this work are potential drugs, so it is necessary to know their solubility in aqueous solutions and partition coefficient between water and 1-octanol, which mimics cell membrane. These factors have significant influence on absorption of drugs by cells and therapeutic effects. Values of water and 1-octanol solubility of tricyclic acyclovir derivatives are shown in Table 1. The acyclovir derivatives can be split into two groups: one, those possessing alkyl or halogen substituents (compounds **1**–**7**), for which aqueous solubility is in the range of 10^−2^ to 10^−3 ^mol·dm^−3^, and the second, with an aromatic group (compounds **8**–**12**) for which the solubility in aqueous solutions is extremely low (10^−4^ to 10^−5^ mol·dm^−3^). Solubilities of the studied compounds in 1-octanol shows the opposite tendency, the solubility is smaller for compounds **1**–**7**, substituted by alkyl or halogen group. Additionally, both solubility of compounds **1**–**12** in water saturated with 1-octanol, and in 1-octanol saturated with water, were determined and compared with the previous results. Compounds for which the aqueous solubility is higher than the solubility in 1-octanol are less soluble if the water is saturated in 1-octanol and more soluble in 1-octanol saturated with water. Solubilities of acyclovir derivatives with aromatic groups, which are more soluble in 1-octanol than in water, are higher if water is saturated with 1-octanol. These dependences are shown in Fig. 2. Similar dependences of aqueous solubility of both groups of the studied compounds were observed at higher temperatures 35 and 45 °C. Aqueous solubilities of compounds **1**–**12** at different temperatures are presented in Table 2 and Fig. 3.

**Table 1:** Solubility of compounds **1**–**12** in water (*C*_w_), water saturated with 1-octanol (*C*_woc_), 1-octanol (*C*_oc_), and 1-octanol saturated with water (*C*_ocw_) at 25 °C

	Compound	*C*_w_ × 10^−4^ (mol·dm^−3^)	*C*_woc_ × 10^−4^ (mol·dm^−3^)	*C*_oc_ × 10^−4^ (mol·dm^−3^)	*C*_ocw_ × 10^−4^ (mol·dm^−3^)
**1**	ACV	80.70 (±1.6)	74.40 (±1.5)	2.12 (±0.18)	3.53 (±0.04)
**2**	Br-ACV	10.30 (±0.8)	8.59 (±0.3)	4.43 (±0.27)	6.89 (±0.40)
**3**	GCV	123.0 (±1.0)	110.0 (±3.0)	1.66 (±0.06)	3.83 (±0.36)
**4**	TACV	30.70 (±0.6)	27.20 (±0.8)	1.40 (±0.03)	4.27 (±0.14)
**5**	6-Me-TACV	187.0 (±3.7)	131.0 (±2.0)	32.4 (±0.03)	35.9 (±0.71)
**6**	2-Br-6-Me-TACV	21.10 (±0.4)	10.51 (±0.05)	2.27 (±0.1)	8.58 (±0.25)
**7**	6-*t*-But-TACV	30.80 (±0.3)	37.80 (±1.1)	159.0 (±2.0)	99.20 (±4.9)
**8**	6-Ph-TACV	2.38 (±0.15)	3.34 (±0.32)	17.80 (±1.2)	15.91 (±0.27)
**9**	6-Ph-Ph-TACV	2.29 (±0.16)	5.83 (±0.01)	20.40 (±1.3)	18.10 (±0.70)
**10**	6-Br-Ph-TACV	3.64 (±0.18)	4.12 (±0.12)	7.80 (±0.08)	6.81 (±0.20)
**11**	6-Ph-O-Me-TACV	0.53 (±0.02)	1.35 (±0.1)	4.03 (±0.06)	3.74 (±0.07)
**12**	6-Napht-TACV	1.66 (± 0.05)	1.91 (±0.04)	7.25 (±0.32)	6.84 (±0.20)

**Table 2:** Aqueous solubility of compounds **1**–**12** at 25, 35, 45 °C

	Compound	*C*_w_ × 10^−4^ (mol·dm^−3^)		
		25 °C	35 °C	45 °C
**1**	ACV	80.70 (±1.6)	162.0 (±8.0)	197.0 (±4.0)
**2**	Br-ACV	10.30 (±0.8)	19.0 (±1.1)	24.9 (±1.2)
**3**	GCV	123.0 (±1.0)	218.0 (±2.0)	229.0 (±3.0)
**4**	TACV	30.70 (±0.6)	59.1 (±2.9)	67.5 (±3.3)
**5**	6-Me-TACV	187.0 (±3.7)	266.0 (±10)	324.0 (±12)
**6**	2-Br-6-Me-TACV	21.10 (±0.4)	23.6 (±0.5)	25.9 (±0.7)
**7**	6-*t*-But-TACV	30.80 (±0.3)	204.0 (±3.0)	319.0 (±5.0)
**8**	6-Ph-TACV	2.38 (±0.15)	5.17 (±0.26)	7.28 (±0.32)
**9**	6-Ph-Ph-TACV	2.29 (±0.16)	5.54 (±0.23)	6.58 (±0. 3)
**10**	6-Br-Ph-TACV	3.64 (±0.18)	8.98 (±0.27)	12.8 (±0.33)
**11**	6-Ph-O-Me-TACV	0.53 (±0.02)	1.44 (±0.07)	4.65 (±0.23)
**12**	6-Napht-TACV	1.66 (±0.05)	2.56 (±0.04)	2.65 (±0.03)

**Fig. 4:**
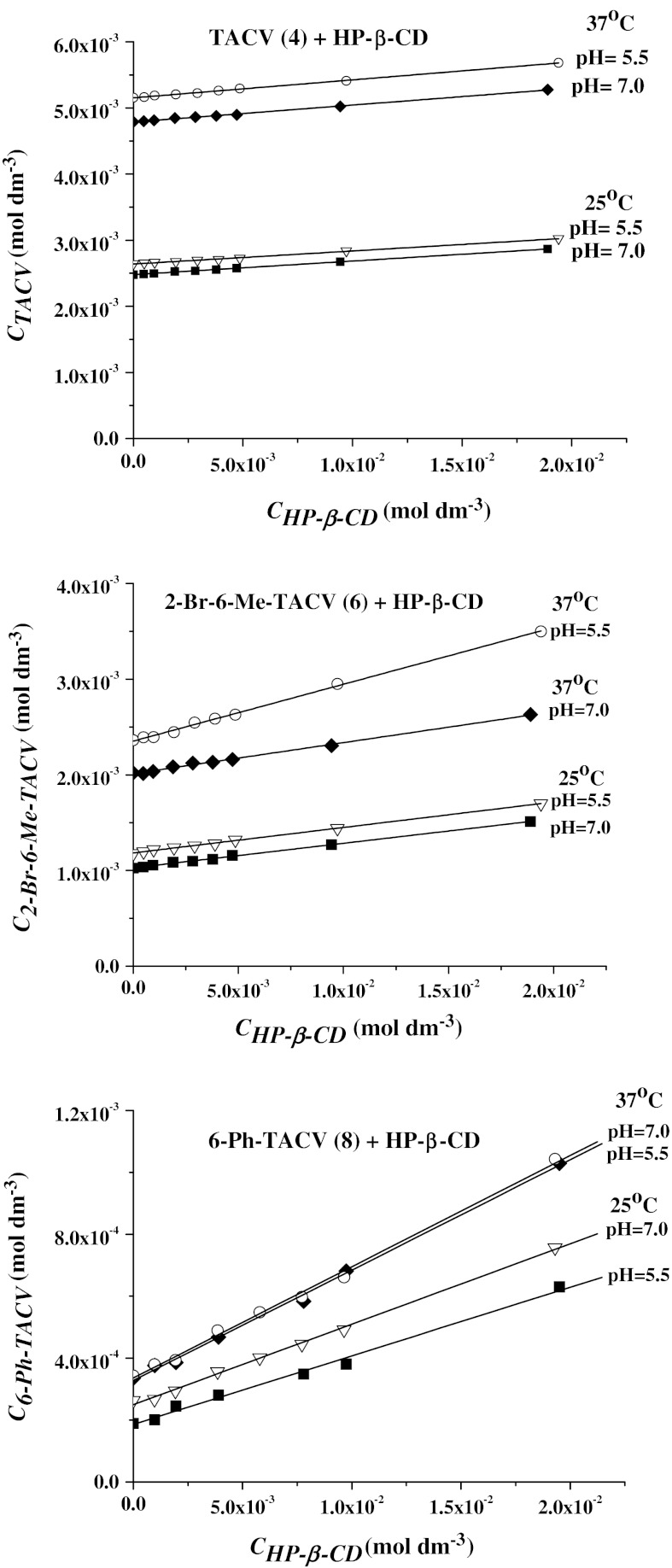
Solubility of tricyclic acylovir derivatives in aqueous buffered solutions (pH = 5.5 and 7.0) containing hydroxypropyl-β-cyclodextrin at 25 and 37 °C

Analysis of the results presented in Tables 1 and 2 indicates that very low aqueous solubility of acyclovir derivatives substituted by aromatic groups, compounds with good antiviral activity towards herpes viruses [15], which is a barrier for their use as drugs. On the other hand, the studied compounds have higher solubility in 1-octanol and in water saturated with 1-octanol than in pure water, which suggests that the drugs may have good permeation properties. The main reason for the low solubility of tricyclic acyclovir derivatives with aromatic groups in water is the high hydrophobicity of these substituents. Use of cyclodextrins, which can include aromatic groups into their cavity, may enhance the aqueous solubility of such compounds. For this purpose we used several cyclodextrins and hydroxypropyl-β-cyclodextrin was chosen as being more efficient for complexating the tricyclic acyclovir derivatives [20]. The investigations were performed, for chosen compounds, in various cyclodextrin HP-β-CD, buffered solutions (pH = 5.5 and 7.0) at 25 and 37 °C. Figure 4 shows the dependence of acyclovir derivative on cyclodextrin concentration for three compounds TACV, 2-Br-6-Me-TaCV and 6-Phe TACV. It was found that the aqueous solubility of the compounds increased linearly with cyclodextrin concentration in the solutions and can be described by the following equation:

2 $$ C_{s} = a + bC_{\text{CD}} $$

where *C*_*s*_ represents the concentration of acyclovir derivative and *C*_CD_ the cyclodextrin concentration.

One exception is acyclovir derivative **7** possessing a *tert*-butyl substituent (6-*t*-But-TACV) where this dependence is not linear over the whole range of cyclodextrin concentrations, showing an A_n_ type of solubility diagram. Values of the correlation parameters *a* and *b* for tricyclic acyclovir derivatives complexed by cyclodextrin are presented in Table 3. The linearity of acyclovir derivative concentration versus cyclodextrin concentration in solutions and slope of line being lower than unity suggest 1:1 stoichiometry of acyclovir derivative—cyclodextrin complexes [29]. The stability constants of the inclusion complexes between HP-β-CD and acyclovir derivatives have been calculated according to the Higuchi–Connors method [30] using:

3 $$ K = \frac{b}{{C_{0} (1 - b)}} $$

where *C*_0_ is the solubility of the sample in solutions without cyclodextrin, obtained by determination of saturated solution concentration of acyclovir derivative solutions. The *a* coefficients in Eq. 2 is the same solubility as *C*_0_, but this value is calculated by the linear regression method. These two values can differ, but in our study are very close to each other.

**Table 3:** Values of the correlation parameters *a* and *b* (Eq. 2) determinated using results of solubility of tricyclic acyclovir derivatives in buffered HP-β-CD solutions

Compound		*t* (°C)	pH = 5.5		pH = 7.0	
			*a*	*b*	*a*	*b*
**4**	TACV	25	2.64 × 10^−3^	1.98 × 10^−2^	2.48 × 10^−3^	2.05 × 10^−2^
		37	5.15 × 10^−3^	2.72 × 10^−2^	4.79 × 10^−3^	2.56 × 10^−2^
**6**	2-Br-6-Me-TACV	25	1.18 × 10^−3^	2.66 × 10^−2^	1.03 × 10^−3^	2.57 × 10^−2^
		37	2.35 × 10^−3^	5.94 × 10^−2^	2.01 × 10^−3^	3.23 × 10^−2^
**7**	6-*t*-But-TACV	25	–	–	–	–
		37	–	–	–	–
**8**	6-Ph-TACV	25	1.86 × 10^−4^	2.22 × 10^−2^	2.50 × 10^−4^	2.60 × 10^−2^
		37	3.27 × 10^−4^	3.57 × 10^−2^	3.36 × 10^−4^	3.60 × 10^−2^
**9**	6-Ph–Ph-TACV	25	2.18 × 10^−4^	2.12 × 10^−2^	2.65 × 10^−4^	2.84 × 10^−2^
		37	5.03 × 10^−4^	2.98 × 10^−2^	5.95 × 10^−4^	3. 89 × 10^−2^
**11**	6-Ph-O-Me-TACV	25	5.23 × 10^−5^	1.00 × 10^−2^	5.68 × 10^−5^	1.06 × 10^−2^
		37	9.95 × 10^−5^	1.41 × 10^−2^	1.37 × 10^−4^	1.30 × 10^−2^
**12**	6-Napht-TACV	25	2.99 × 10^−5^	0.88 × 10^−2^	3.05 × 10^−5^	0.94 × 10^−2^
		37	3.37 × 10^−5^	0.93 × 10^−2^	4.29 × 10^−5^	1.29 × 10^−2^

**Fig. 5:**
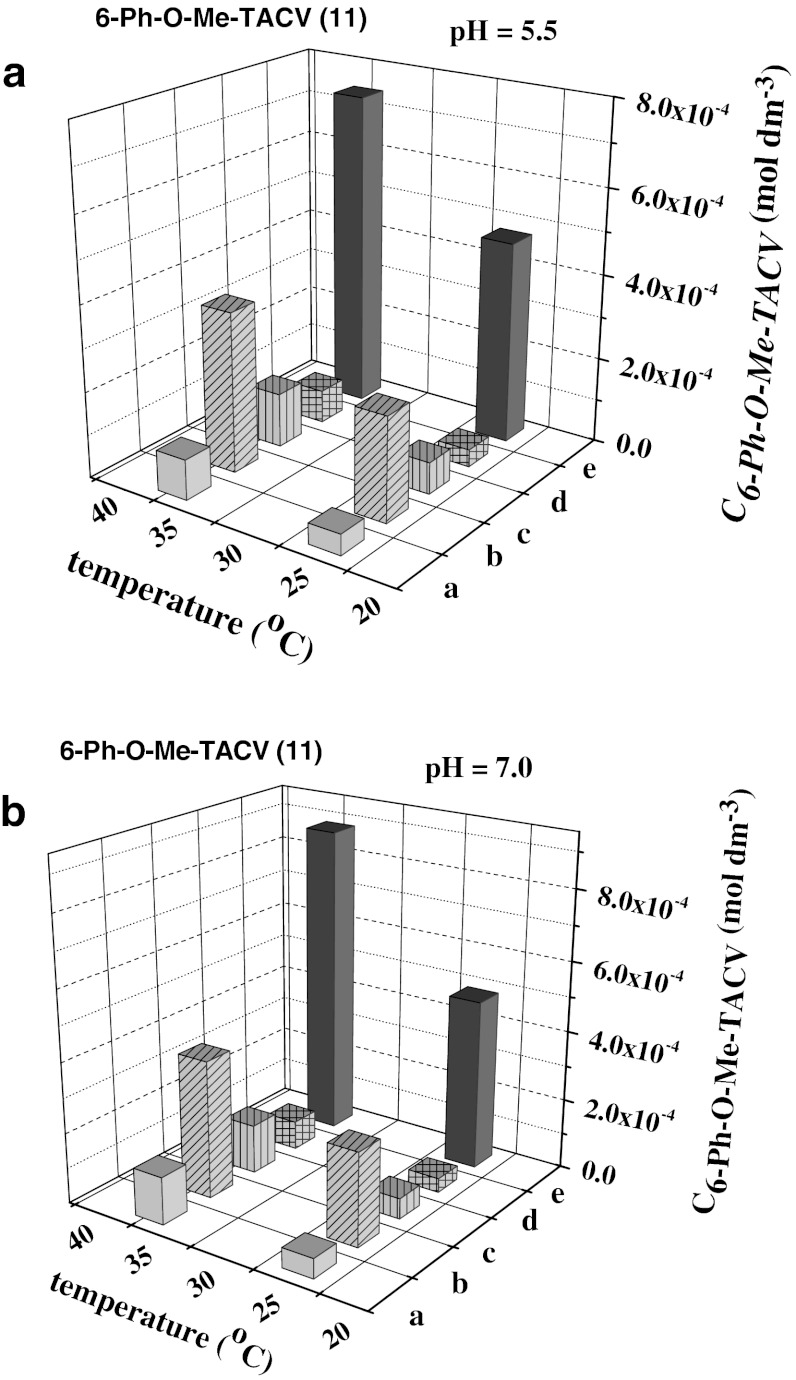
Comparison of solubility of compound **11** (6-Ph-O-Me-TACV**)** in buffered aqueous solutions at pH = 5.5 (**A**) and 7.0 (**B**) (*a*), the same buffered solutions containing 2 × 10^−2^ (mol·dm^−3^) HP-β-CD (*b*), buffered solutions saturated with 1-octanol (*c*) and containing 2 × 10^−2^ (mol·dm^−3^) HP-β-CD (*d*), and in 1-octanol saturated with buffer (*e*)

Table 4 presents values of stability constants of cyclodextrin inclusion complexes and enhancement of aqueous solubility connected with the presence of cyclodextrin in the solutions. The highest values of stability constants and 3–7 fold enhancement of solubility are observed for compounds possessing aromatic groups, which may be bound inside the cyclodextrin cavity. ^1^H NMR measurements confirm these conclusions [19]. Comparison of the solubilities of acyclovir derivative **11** (6-Ph-O-Me-TACV) in buffer solutions (pH = 5.5 and 7.0), buffer containing 2 × 10^−2^ mol·dm^−3^ HP-β-CD, buffer saturated with 1-octanol and containing 2 × 10^−2^ mol·dm^−3^ HP-β-CD, and 1-octanol saturated with buffer indicates that the highest concentrations of 6-Ph-O-Me-TACV are observed in 1-octanol and cyclodextrin solutions (see Fig. 5 and Table 5). The low concentration of acyclovir derivative in buffer saturated with 1-octanol and containing cyclodextrin is connected with complexation of 1-octanol in the cyclodextrin cavity that prevents complexation of 6-Ph-O-Me-TACV. This conclusion is important for possible pharmaceutical usage of these derivatives. Complexation of such drugs by cyclodextrin enhances their aqueous solubility but in contact with 1-octanol, which mimics the cell membrane, the drug is easily released from the cyclodextrin complex as a result of competitive complexation of 1-octanol. The values of partition coefficient of these compounds in 1-octanol-water calculated from extraction experiments indicate good permeability of tricyclic acyclovir derivatives substituted with aromatic groups, so the released drug can penetrate the cell membrane.

**Table 4:** Stability constants of hydroxypropyl-β-complexes with tricyclic acyclovir derivatives and relative enhancement of aqueous solubility, *C*_s_/*C*_o_, of the derivatives in cyclodextrin buffered solutions at pH = 5.5 and 7.0 and two temperatures 25 and 37 °C. The concentration of cyclodextrin is 2 × 10^−2^ (mol·dm^−3^)

Compound		pH = 5.5				pH = 7			
		*K*_s_ (dm·mol^−1^)		*C*_s_/*C*_o_		*K*_s_ (dm·mol^−1^)		*C*_s_/*C*_o_	
		25 °C	37 °C	25 °C	37 °C	25 °C	37 °C	25 °C	37 °C
**4**	TACV	8.0 (±1)	6.0 (±1)	1.11	1.1	8.0 (±1)	6.0 (±1)	1.1	1.1
**6**	2-Br-6-Me-TACV	27 (±2)	26 (±2)	1.4	1.5	26 (±2)	17 (±1)	1.5	1.3
**7**	6-*t*-But-TACV	–	–	1.5	1.6	–	–	1.2	1.3
**8**	6-Ph-TACV	122 (±2)	113 (±10)	3.3	3.0	107 (±8)	105 (±6)	3.1	3.0
**9**	6-Ph-Ph-TACV	100 (±8)	61 (±5)	2.8	2.1	110 (±6)	68 (±4)	3.0	2.3
**11**	6-Ph-O-Me-TACV	213 (±6)	144 (±5)	5.1	3.8	189 (±6)	96 (±4)	4.8	2.9
**12**	6-Napht-TACV	297 (±7)	279 (±9)	7.7	6.4	311 (±9)	305 (±9)	8.1	6.9

**Table 5:** Comparison of the solubility of compound **11** (6-Ph-O-Me-TACV) in buffered aqueous solutions at pH = 5.5 and 7.0, the same buffered solutions containing 2 × 10^−2^ (mol·dm^−3^) HP-β-CD, buffered solutions saturated with 1-octanol and containing 2 × 10^−2^ (mol·dm^−3^) HP-β-CD, and in 1-octanol saturated with buffer

Solution	*C*_6-Ph-O-Me-TACV_ × 10^−5^ (mol·dm^−3^)			
	pH = 5.5		pH = 7.0	
	25 °C	37 °C	25 °C	37 °C
Buffer	4.82 (±0.15)	9.94 (±0.1)	5.50 (±0.23)	13.6 (±0.31)
Buffer + HP-β-CD	24.4 (±0.82)	37.4 (±1.1)	26.5 (±95)	39.1 (±91)
Buffer saturated with 1-octanol	7.42 (±0.05)	12.7 (±0.1)	5.87 (±0.18)	13.9 (±0.21)
Buffer saturated with 1-octanol + HP-β-CD	4.03 (±0.12)	7.70 (±0.21)	4.03 (±0.2)	8.06 (±0.32)
1-Octanol saturated with buffer	47.3 (±0.5)	74.1 (±0.41)	48.5 (±1.1)	88.2 (±2.3)

**Fig. 6:**
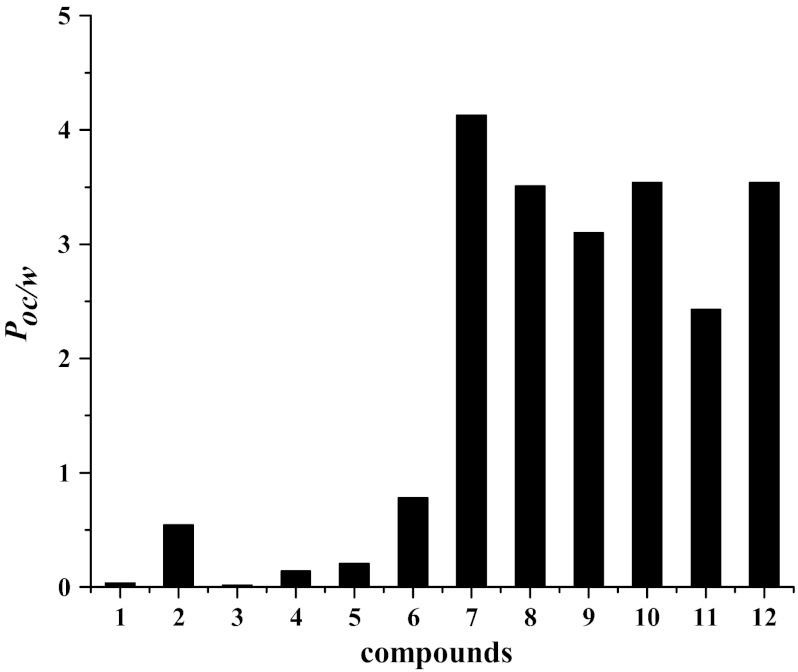
Partition coefficient 1-octanol–water of compounds **1**–**12** at 25 °C

**Fig. 7:**
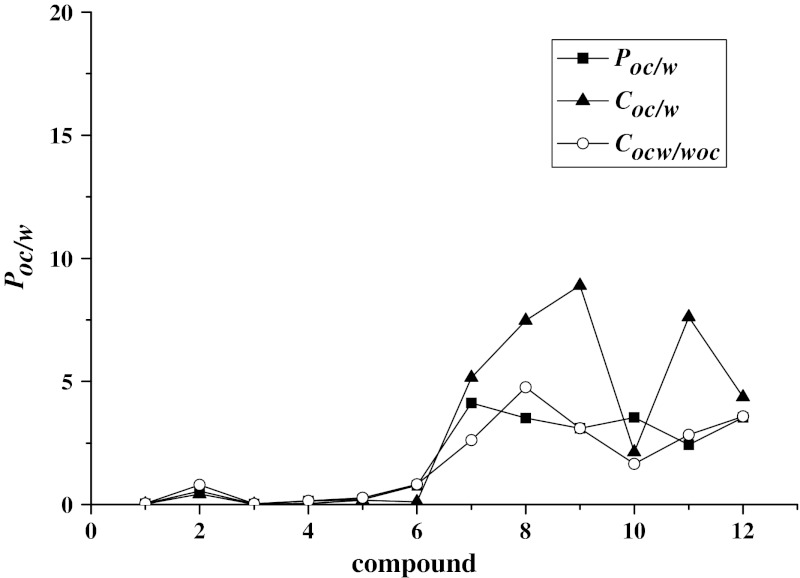
Comparison of 1-octanol-water partition coefficient (*P*_oc/w_) of compounds **1**–**12** with ratios of solubility of these compounds in 1-octanol and water (*C*_oc_/*C*_w_) or 1-octanol saturated with water, and water saturated with 1-octanol (*C*_ocw_/*C*_woc_) at 25 °C

The 1-octanol–water partition coefficients, for all compounds, are presented in Fig. 6 and Table 6. Table 6 contains, for comparison, values of the ratios: solubility in 1-octanol and in water (*C*_oc_/*C*_w_) as well as solubility in water saturated with 1-octanol and in 1-octanol saturated with water (*C*_ocw_/*C*_woc_). The partition coefficients have the same sequence as the solubilities in 1-octanol, higher values were observed for compounds with aromatic groups (see Fig. 7). The same tendency were observed for the *C*_ocw_/*C*_woc_ ratio, which may be used to envisage permeation possibilities of potential drugs if extraction experiments are not performed. Enhancement of the aqueous solubility of acyclovir derivatives can be treated as a first step for further study in preparation of new drugs containing these compounds.

**Table 6:** Partition coefficient 1-octanol–water (*P*_oc/w_) of compounds **1**–**12**, and solubility ratios of these compounds in 1-octanol and water (*C*_oc_ */C*_w_ *),* in 1-octanol saturated with water and water saturated with 1-octanol (*C*_ocw_ */C*_woc_)

	Compound	*P*_oc/w_	*C*_oc_/*C*_w_	*C*_ocw_/*C*_woc_
**1**	ACV	0.036 (±0.001)	0.026	0.048
**2**	Br-ACV	0.544 (±0.09)	0.430	0.802
**3**	GCV	0.017 (±0.001)	0.013	0.035
**4**	TACV	0.138 (±0.001)	0.046	0.157
**5**	6-Me-TACV	0.205 (±0.005)	0.173	0.274
**6**	2-Br-6-Me-TACV	0.780 (±0.009)	0.108	0.816
**7**	6-t-But-TACV	4.13 (±0.04)	5.16	2.62
**8**	6-Ph-TACV	3.51 (±0.29)	7.48	4.76
**9**	6-Ph–Ph-TACV	3.10 (±0.08)	8.90	3.10
**10**	6-Br-Ph-TACV	3.54 (±0.17)	2.14	1.65
**11**	6-Ph-O-Me-TACV	2.43 (±0.22)	7.62	2.84
**12**	6-Napht-TACV	3.54 (±0.01)	4.37	3.58

**Fig. 8:**
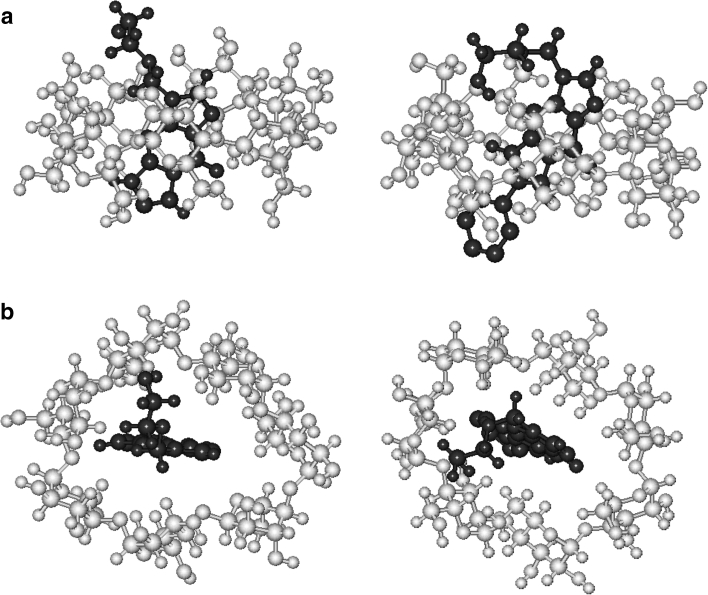
Structures of optimized structures of β-cyclodextrin complexes with tricyclic acyclovir derivatives TACV and 6-Ph-TACV. Side view (**a**) and upper view (**b**)

In order to know possible structures of the cyclodextrin complexes with tricyclic acyclovir, we performed a series of molecular mechanics and molecular dynamics calculations. Because of the lack of a well defined structure of hydroxypropyl-β-cyclodextrin, β-cyclodextrin has been taken for the calculations. Calculations were performed assuming vacuum surroundings. Based of them, the energy of complexation and surface changes of cyclodextrin and acyclovir derivative molecules during complexation were determined. Results of the calculations are presented in Table 7. Looking at the results it is seen that the energy of complexation is connected with the stability constants of the complexes and changes in surfaces with enhancement of aqueous solubility. They show how deeply the hydrophobic group is included in the cyclodextrin cavity and why it cannot interact with water in solutions. Optimized structures of β-cyclodextrin complexes with two acyclovir derivatives TACV and 6-Ph-TACV are presented in Fig. 8.

**Table 7:** Energy of complexation and changes of β-cyclodextrin, acyclovir derivatives and their inclusion complex surfaces determinated by optimization of the complex structure using molecular mechanics and molecular dynamics methods

	Compound	Δ*E*_com_^a^ (kJ·mol^−1^)	Δ*S*_com_^b^ (Å^2^)	Δ*S*_s_^c^ (Å^2^)	Δ*S*_CD_^d^ (Å^2^)
**4**	TACV	−180.00	−377.2	278.9	179.8
**6**	2-Br-6-Me-TACV	−173.42	−434.8	302.9	214.3
**7**	6-*t*-But-TACV	−186.90	−517.9	342.4	243.0
**8**	6-Ph-TACV	−184.36	−486.0	325.5	241.9
**9**	6-Ph-Ph-TACV	−181.31	−512.3	338.6	261.7
**11**	6-Ph-O-Me-TACV	−202.22	−491.1	345.0	253.1
**12**	6-Naft- TACV	−209.91	−536.1	362.4	249.4

^a^ $\Updelta E_{\text{com}} = E_{\text{com}} - (E_{\text{s}} + E_{\text{CD}} )$, Δ*E*_com_—energy of complexation, *E*_com_—calculated energy of cyclodextrin inclusion complex with acyclovir derivative, *E*_s_—calculated energy of acyclovir derivative molecule, *E*_CD_—calculated energy of cyclodextrin molecule

^b^ $\Updelta S_{\text{com}} = S_{\text{com}} - (S_{\text{s}} + S_{\text{CD}} )$, Δ*S*_com_—change of surfaces during complexation process, *S*_com_—surface of complex, *S*_s_ –surface of free acyclovir derivative molecule, *S*_CD_—surface of free cyclodextrin molecule

^c^ $\Updelta S_{\text{S}} = S_{{{\text{s}}1}} - S_{{{\text{s}}2}}$, Δ*S*_s_—difference between surfaces *S*_s1_ and *S*_s2_ of acyclovir derivative complexed by cyclodextrin, where *S*_s2_ is the surface of acyclowir derivative in the presence of cyclodextrin and *S*_s1_ in the absence of cyclodextrin

^d^Surfaces of cyclodextrin molecule in the complex, Δ*S*_CD_, Δ*S*_CD1_ and Δ*S*_CD2_, were calculated in the same manner as surfaces of acyclovir derivatives

## Conclusions

The comparison of solubility of tricyclic acyclovir derivatives in water and buffered solutions of HP-β-CD suggests that addition of cyclodextrin enhances the aqueous solubility of the studied compounds by complexation of hydrophobic substituents in the cyclodextrin cavity. Values of stability constants, which are in the range 10–300, give the opportunity to easily release the compounds in the human body. Partition coefficients in the range 2.5–4.1 for derivatives with an aromatic group suggest good absorption in body cells.
